# Supplementary material for: Proximal protein landscapes of the type I interferon signaling cascade reveal negative regulation by PJA2
Source: Nat Commun. 2024 May 27;15:4484. doi: 10.1038/s41467-024-48800-5 (PMC11130243; doi:10.1038/s41467-024-48800-5)
Supplement: Supplementary file 1 — Supplementary Information [file 41467_2024_48800_MOESM1_ESM.pdf]

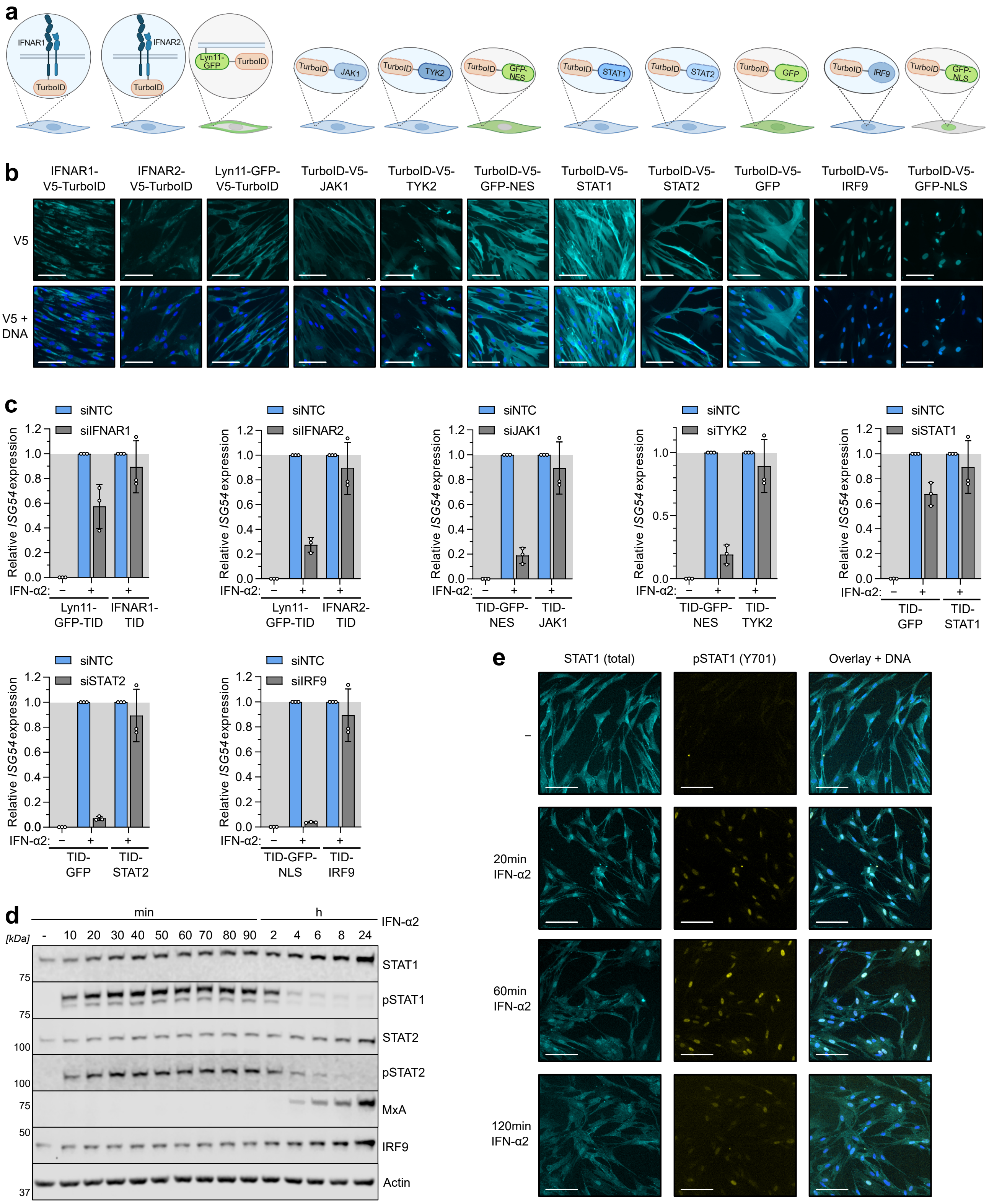

Supplementary Fig. 1: Validation of TurboID-based type I IFN signaling constructs. a Schematic representation of the TurboID-tagged constructs used for proximity labeling. Each construct has a V5

tag (not depicted) that serves as a linker between TurboID and the protein of interest. **b** Immunofluorescence (IF) imaging (representative of n=3 images) of the intracellular localization of each TurboID-tagged construct in the transduced MRC-5/hTERT cells using anti-V5 antibody. DNA was stained with DAPI. Scale bars represent 100 µm. **c** *ISG54* mRNA expression as determined by RT-qPCR following ± 4 h IFN-α2 stimulation (1000 IU/mL). The indicated transduced MRC-5/hTERT cells had previously been reverse transfected with siRNAs targeting the 3'untranslated region of the indicated gene or a non-targeting siRNA (NTC). Data are normalized to *GAPDH* expression levels in the same sample and made relative to the NTC + IFN-α2 condition. Bars represent means ± SDs from n=3 independent experiments conducted in technical duplicates. **d** MRC-5/hTERT cells were stimulated with 1000 IU/mL IFN-α2 for the indicated times. Total cell lysates were analyzed by SDS-PAGE and immunoblotting for the indicated proteins in n=2 independent experiments. **e** IF imaging (representative of n=3 images) of total and phosphorylated STAT1 in MRC-5/hTERT cells following stimulation with 1000 IU/mL IFN-α2 for the indicated times. DNA was stained with DAPI. Scale bars represent 100 µm.

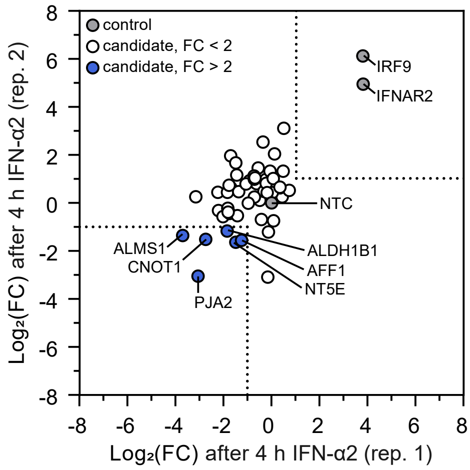

Supplementary Fig. 2: siRNA screening identifies proximal proteins that functionally regulate type I IFN antiviral activity. Log_2(FC) in VSV-GFP replication (AUC values) after 4 h IFN-α2 stimulation of MRC-5 cells previously transfected with siRNAs targeting the indicated genes. Log2(FC) is relative to VSV-GFP replication in the NTC condition. n=2 independent replicates are plotted. The dotted lines indicate a 2-fold change from the VSV-GFP replication in the NTC condition.

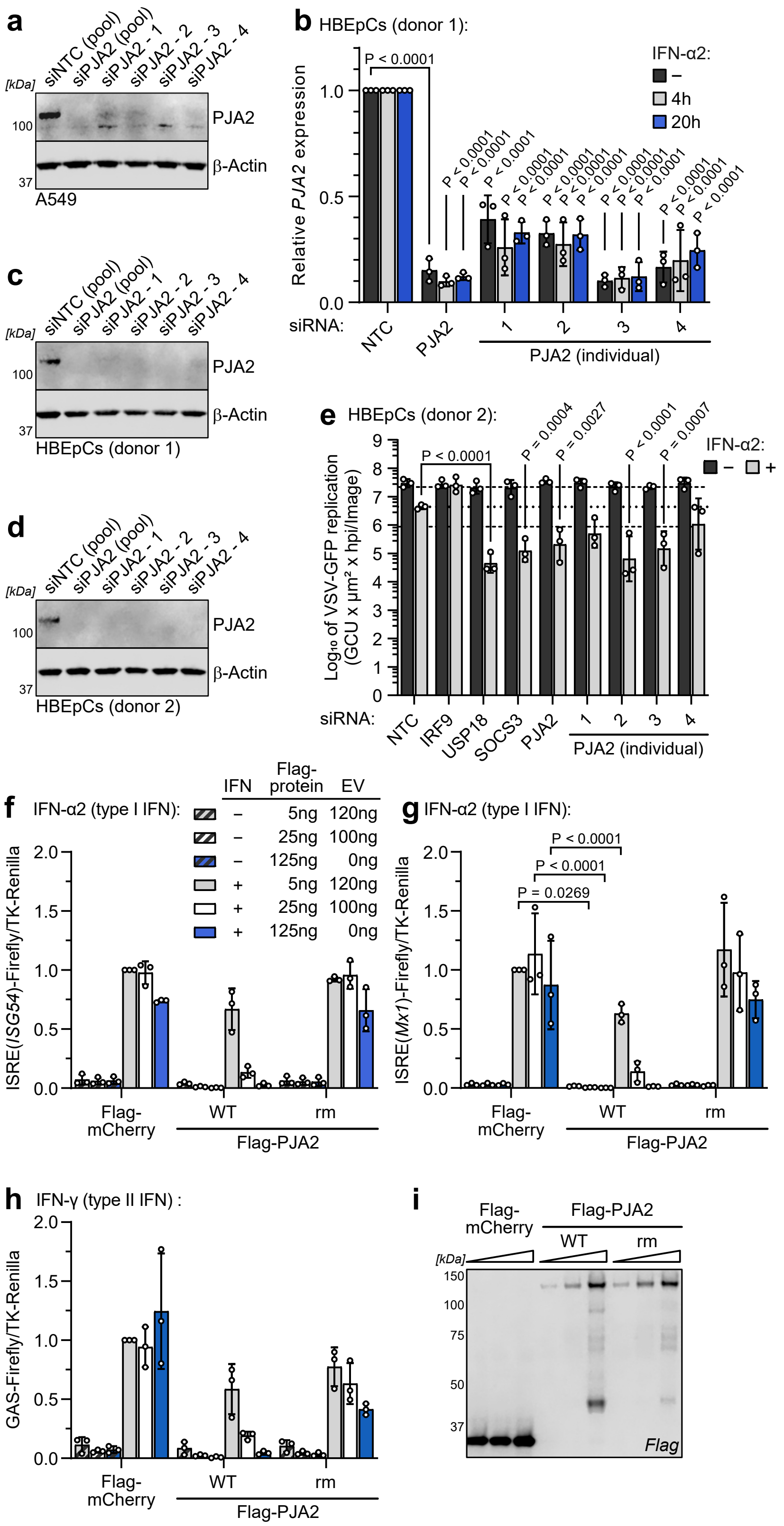

Supplementary Fig. 3: PJA2 depletion is efficient with both pooled and single siRNAs, and PJA2 negatively regulates type I IFN signaling in multiple systems. a Representative immunoblot from the experiments shown in main Fig. 6a to show the expression levels of PJA2. Data are representative of n=3 independent experiments. **b**, **c** Representative *PJA2* mRNA expression (**b**) and protein immunoblot (**c**) from the experiments shown in Fig. 6b, c. Data are representative of n=3 independent experiments. RT-qPCR data are normalized to *GAPDH* expression levels in the same sample and made

relative to the NTC condition. Bars represent means ± SDs from n=3 independent experiments. Statistically significant P values are shown compared to the similarly stimulated NTC sample and were determined by two-way ANOVA and Dunnett's multiple comparisons. **d** Representative immunoblot from the experiment shown in panel e to show the expression levels of PJA2. **e** VSV-GFP assay showing viral replication in the indicated siRNA-transfected HBEpCs (donor 2) ± 16 h IFN-α2 (10 IU/mL) stimulation. Total GFP levels were calculated from AUC values for VSV-GFP replication during the course of the experiment. Means ± SDs from n=3 independent experiments are shown. The dotted line indicates VSV-GFP replication in the NTC condition after IFN-α2 stimulation, and the dashed lines indicate a 5-fold change. P values compared to the NTC sample were determined by two-way ANOVA and Šídák's multiple comparisons. Non-significant values (P > 0.05) are not shown. **f** - **h** HEK293T cells were co-transfected with ISRE(*ISG54*)-Firefly (**f**, same experiment as main Fig. 6j), ISRE(*Mx1*)-Firefly (**g**) or GAS-Firefly (**h**, same experiment as main Fig. 6k) plasmids together with a TK-Renilla control and 5, 25, or 125 ng of plasmid expressing Flag-tagged PJA2-WT, PJA2-rm or mCherry. The total plasmid DNA transfected was kept constant by addition of the appropriate amount of empty vector (EV). Following 16 h of 100 IU/mL IFN-α2 (**f**), 100 IU/mL IFN-α2 (**g**), or 1000 IU/mL IFN-γ (**h**) stimulation, luciferase activities were determined and normalized to the 5 ng mCherry control condition post IFN-α2 stimulation. Bars represent mean ± SD values from n=3 independent experiments. P values were determined by two-way ANOVA and Dunnett's multiple comparisons. Non-significant values (P > 0.05) are not shown. **i** Representative immunoblot from the experiments shown in Fig. 6j, k and Supplementary Fig. 3f-h to show the expression levels of the indicated Flag-tagged proteins. Data are representative of n=3 independent experiments. All exact P values and details on statistical tests are provided in the Source Data file.

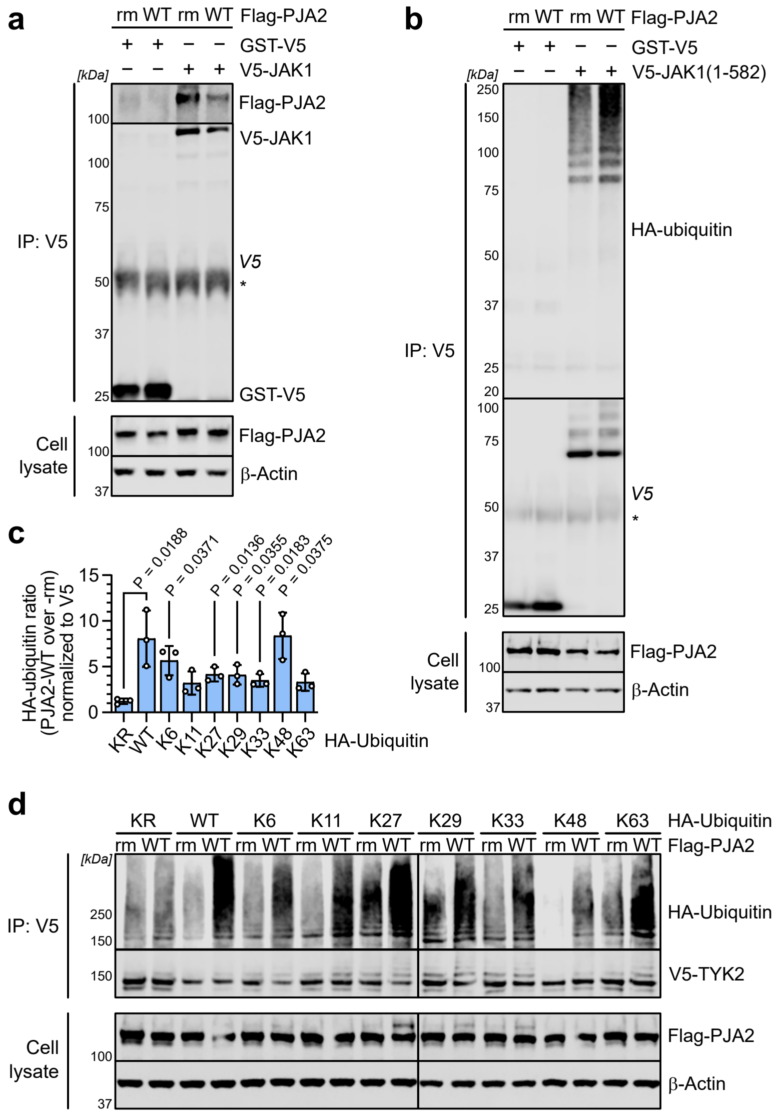

Supplementary Fig. 4: PJA2 interacts with JAK1, ubiquitinates JAK1(1-582), and ubiquitinates TYK2 with diverse ubiquitin chains. a HEK293T cells were transfected with plasmids expressing V5-JAK1 or GST-V5 together with Flag-PJA2-WT or PJA2-rm to generate cell lysates prior to anti-V5 immunoprecipitation (IP). Cell lysate and IP fractions were then analyzed by SDS-PAGE and immunoblotting for the indicated proteins. Data are representative of n=3 independent experiments. * indicates IgG heavy chain. **b** HEK293T cells were co-transfected with V5-JAK1(1-582) or GST-V5 together with Flag-tagged PJA2-WT or PJA2-rm and HA-ubiquitin. Cells were lysed in a denaturing buffer containing 2 % SDS which was diluted to 0.7 % SDS prior to anti-V5 IP. Cell lysate and IP fractions were analyzed by SDS-PAGE and immunoblotting for the indicated proteins. Data are representative of n=3 independent experiments. * indicates IgG heavy chain. **c** Quantification of the HA-ubiquitin signal normalized to the V5 signal of the same IP from the experiments shown in panel **d**, with HA-ubiquitin/V5 quantities for the PJA2-WT condition made relative to the PJA2-rm condition. Bars represent means ± SDs from n=3 independent replicates. P values were determined by two-sided unpaired t test with Welch's correction. **d** HEK293T cells were co-transfected with V5-TYK2 together with Flag-tagged PJA2-WT or PJA2-rm and different HA-ubiquitin mutants. Cells were lysed in a denaturing buffer containing 2 % SDS which was diluted to 0.7 % SDS prior to anti-V5 IP. Cell lysate and IP fractions were analyzed by SDS-PAGE and immunoblotting for the indicated proteins. Data are representative of n=3 independent experiments. All exact P values and details on statistical tests are provided in the Source Data file.

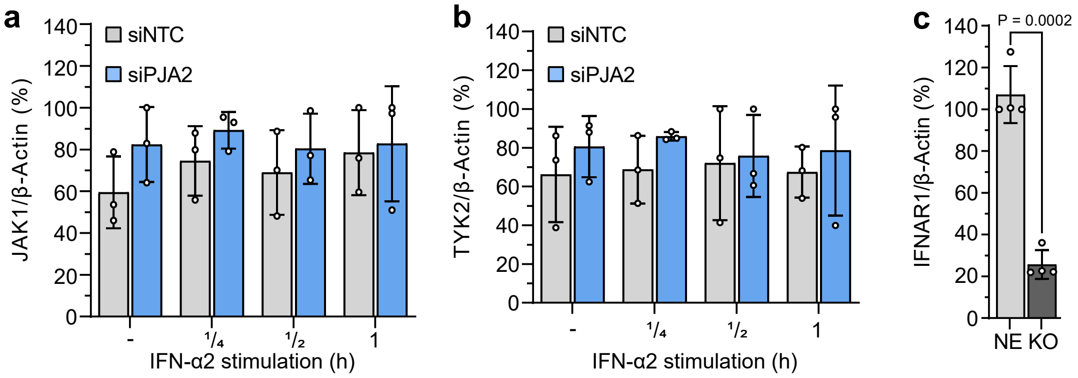

Supplementary Fig. 5: PJA2 does not affect JAK1 or TYK2 protein levels. a, **b** Immunoblot quantification of JAK1 (**a**) or TYK2 (**b**) protein levels normalized to β-actin protein levels in replicate experiments of that shown in main Fig. 8a. Quantifications were made relative to the highest value in each experiment. Bars represent means ± SDs from n=3 independent experiments. All P values were non-significant (P > 0.05), are not displayed, and were determined by two-way ANOVA and Šídák's multiple comparisons. **c** Immunoblot quantification of IFNAR1 protein expression normalized to β-actin protein expression and made relative to one of the NE control cell clones in replicates of what is shown in main Fig. 8c. Bars represent means ± SDs from n=2 independent experiments with n=2 independent NE and KO cell lines (n=4 total). Statistical significance was determined by two-sided unpaired t test with Welch's correction. All exact P values and details on statistical tests are provided in the Source Data file.

Supplementary Table 1: LentiCRISPR oligonucleotides

Name	Forward primer	Reverse primer
**sgPJA2-1**	CACCGAATGGACCAAGAATCTGGTA	AAACTACCAGATTCTTGGTCCATTC
**sgPJA2-2**	CACCGCGTGGAAGGTAGTAGATACC	AAACGGTATCTACTACCTTCCACGC
**sgPJA2-3**	CACCGATCAACTTGATCCAAAGGAC	AAACGTCCTTTGGATCAAGTTGATC

Supplementary Table 2: crRNA

Target	Sequence
**TYK2**	ACCUGCGGAAGACGUUCCGAGUUUUAGAGCUAUGCU

Supplementary Table 3: NGS primers

Target	Forward primer	Reverse primer
**TYK2**	CTTTCCCTACACGACGCTCTTCCGA<br>TCTCCCAGCTTCAAGGACTGCAT	GACTGGAGTTCAGACGTGTGCTCTTC<br>CGATCTCACTGTCCCGGATGTAGCAG

Supplementary Table 4: Qiagen FlexiTube siRNAs

Target	Catalog Number	Gene Accession
**AllStars negative control**	1027280	
**IFNAR1**	SI00013195	NM_000629
**IFNAR2**	SI05005896	NM_207585
**JAK1**	SI04991378	NM_002227
**TYK2**	SI02223221	NM_003331
**STAT1**	SI05078556	NM_007315, NM_139266
**STAT2**	SI05020876	NM_005419, NM_198332
**IRF9**	SI00084364	NM_006084
**PJA2 – 1**	SI00108325	NM_014819
**PJA2 – 2**	SI00108332	NM_014819
**PJA2 – 3**	SI00108339	NM_014819
**PJA2 – 4**	SI00108346	NM_014819

Supplementary Table 5: Horizon Discovery ON-TARGETplus siRNA SMARTpools

Target	Catalog Number	Gene Accession
**ON-TARGETplus Non-targeting Control**	D-001810-10	
**AFF1**	L-020074-02	NM_001166693
**AIM1**	L-024709-01	NM_001624
**ALDH1B1**	L-008254-00	NM_000692
**ALMS1**	L-012889-00	NM_015120
**ANKRD17**	L-013554-01	NM_198889
**APC**	L-003869-00	NM_000038
**APPL1**	L-005138-00	NM_012096
**ARFGEF1**	L-012207-00	NM_006421
**ASCC2**	L-016458-02	NM_032204
**CALCOCO1**	L-007038-01	NM_020898
**CNOT1**	L-015369-01	NM_206999
**CTPS1**	L-006644-00	NM_001905
**DNAJA2**	L-012104-00	NM_005880
**DNMBP**	L-026304-01	NM_015221
**ECD**	L-019678-00	NM_007265
**EEF1D**	L-011648-01	NM_001130056
**EIF4E2**	L-019870-01	NM_004846
**FAM175B**	L-016146-01	NM_032182
**GIGYF2**	L-013918-01	NM_015575
**HECTD1**	L-007188-00	NM_015382
**HSP90AB1**	L-005187-00	NM_007355
**HTRA1**	L-006009-00	NM_002775
**IFNAR2**	L-015411-00	NM_207584
**IGF2BP3**	L-003976-00	NM_006547
**IL6ST**	L-005166-00	NM_175767
**IRF9**	L-020858-00	NM_006084
**KLC1**	L-019482-00	NM_182923
**KLC2**	L-014218-00	NM_022822
**LRBA**	L-012751-00	NM_006726
**LRCH4**	L-011321-01	NM_002319
**MADD**	L-004429-00	NM_130474
**MAP1A**	L-013482-00	NM_002373
**MFSD10**	L-016015-01	NM_001120
**MTCL1**	L-023376-01	NM_015210
**MYCBP2**	L-006951-00	NM_015057
**NT5E**	L-008217-00	NM_002526
**OSMR**	L-008050-00	NM_003999
**PCCA**	L-008965-00	NM_000282
**PCM1**	L-005165-00	NM_006197
**PGM3**	L-013912-01	NM_015599

**PHLDB2**	L-016702-01	NM_145753
**PJA2**	L-006916-00	NM_014819
**PLEKHG2**	L-023690-00	NM_022835
**RAB1B**	L-008958-01	NM_030981
**RIPK1**	L-004445-00	NM_003804
**SOCS3**	L-004299-00	NM_003955
**STAT6**	L-006690-00	NM_003153
**TBC1D2**	L-020463-01	NM_018421
**TEX2**	L-017117-02	NM_018469
**TMEM131**	L-022532-02	NM_015348
**USP18**	L-004236-00	NM_017414
**USP9X**	L-006099-00	NM_021906
**WNK1**	L-005362-02	NM_014823
**WWC2**	L-016585-02	NM_024949

Supplementary Table 6: qPCR primers

Target	Forward primer	Reverse primer
**ISG54**	GCGTGAAGAAGGTGAAGAGG	GCAGGTAGGCATTGTTTG
**MX1**	AGACAAGGTTGTGGACGTGG	TTCCTCCAGCAGATCCCTGA
**GAPDH**	CTGGCGTCTTCACCACCATGG	CATCACGCCACAGTTTCCCGG
**PJA2**	CATTTCGGAATCTTCTGCGGC	CATCACCAGCCCGACCTAAG
