# Supplementary material for: Proximal protein landscapes of the type I interferon signaling cascade reveal negative regulation by PJA2
Source: Nat Commun. 2024 May 27;15:4484. doi: 10.1038/s41467-024-48800-5 (PMC11130243; doi:10.1038/s41467-024-48800-5)
Supplement: Supplementary file 2 — Reporting Summary [file 41467_2024_48800_MOESM2_ESM.pdf]

nature portfolio

Corresponding author(s): Hale

Last updated by author(s): Apr 23, 2024

Reporting Summary

Nature Portfolio wishes to improve the reproducibility of the work that we publish. This form provides structure for consistency and transparency in reporting. For further information on Nature Portfolio policies, see our Editorial Policies and the Editorial Policy Checklist.

Statistics

For all statistical analyses, confirm that the following items are present in the figure legend, table legend, main text, or Methods section.

n/a	Confirmed	
☐	☒	The exact sample size (*n*) for each experimental group/condition, given as a discrete number and unit of measurement
☐	☒	A statement on whether measurements were taken from distinct samples or whether the same sample was measured repeatedly
☐	☒	The statistical test(s) used AND whether they are one- or two-sided<br>*Only common tests should be described solely by name; describe more complex techniques in the Methods section.*
☒	☐	A description of all covariates tested
☒	☐	A description of any assumptions or corrections, such as tests of normality and adjustment for multiple comparisons
☐	☒	A full description of the statistical parameters including central tendency (e.g. means) or other basic estimates (e.g. regression coefficient) AND variation (e.g. standard deviation) or associated estimates of uncertainty (e.g. confidence intervals)
☐	☒	For null hypothesis testing, the test statistic (e.g. *F*, *t*, *r*) with confidence intervals, effect sizes, degrees of freedom and *P* value noted<br>*Give P values as exact values whenever suitable.*
☒	☐	For Bayesian analysis, information on the choice of priors and Markov chain Monte Carlo settings
☒	☐	For hierarchical and complex designs, identification of the appropriate level for tests and full reporting of outcomes
☒	☐	Estimates of effect sizes (e.g. Cohen's *d*, Pearson's *r*), indicating how they were calculated

Our web collection on statistics for biologists contains articles on many of the points above.

Software and code

Policy information about availability of computer code

Data collection	Immunoblot images were developed with the Image Studio Lite Quantification software (LI-COR Biosciences). Mass spectrometry data was obtained on a Q Exactive mass spectrometer (Thermo Fisher Scientific). qPCR data was measured on the ABI7300 Real-Time PCR system (Applied Biosystems). Immunofluerescence was imaged with a Leica DM IL LED microscope (Leica Microsystems) using the LasX software.
Data analysis	GraphPad Prism 9 was used.<br>Proximity labeling: MS raw data was converted into mzML files with MSConvert (ProteoWizard software package), proteins were identified using FragPipe (v13.0) and MSFragger, and enrichements were analyzed with SAINTexpress: https://reprint-apms.org/.<br>Functional enrichment gene ontology terms were analyzed with DAVID: https://david.ncifcrf.gov/.

For manuscripts utilizing custom algorithms or software that are central to the research but not yet described in published literature, software must be made available to editors and reviewers. We strongly encourage code deposition in a community repository (e.g. GitHub). See the Nature Portfolio guidelines for submitting code & software for further information.

Data

Policy information about availability of data

All manuscripts must include a data availability statement. This statement should provide the following information, where applicable:

- Accession codes, unique identifiers, or web links for publicly available datasets
- A description of any restrictions on data availability
- For clinical datasets or third party data, please ensure that the statement adheres to our policy

All data are available within the paper and its supplementary information. The mass spectrometry proteomics data have been deposited to the ProteomeXchange Consortium via the PRIDE partner repository with the dataset identifier PXD045482. Source data are provided with this paper.

Research involving human participants, their data, or biological material

Policy information about studies with human participants or human data. See also policy information about sex, gender (identity/presentation), and sexual orientation and race, ethnicity and racism.

Reporting on sex and gender	The study does not include research on human participants or their data.
Reporting on race, ethnicity, or other socially relevant groupings	The study does not include research on human participants or their data.
Population characteristics	The study does not include research on human participants or their data.
Recruitment	The study does not include research on human participants or their data.
Ethics oversight	No ethics approval was deemed necessary for the study.

Note that full information on the approval of the study protocol must also be provided in the manuscript.

Field-specific reporting

Please select the one below that is the best fit for your research. If you are not sure, read the appropriate sections before making your selection.

☒ Life sciences ☐ Behavioural & social sciences ☐ Ecological, evolutionary & environmental sciences

For a reference copy of the document with all sections, see nature.com/documents/nr-reporting-summary-flat.pdf

Life sciences study design

All studies must disclose on these points even when the disclosure is negative.

Sample size	No sample size was determined prior to the study. The number of samples measured is in-line with field-specific practices.
Data exclusions	No data was excluded from the experiments and the analysis.
Replication	The number of replicates for each experiment is indicated in the appropriate legends. No standard number of replicates was determined prior to the study. The number of replicates performed is in-line with field-specific practices.
Randomization	Samples were not randomised during the study.
Blinding	Investigators were not blinded to sample identity during the study.

Reporting for specific materials, systems and methods

We require information from authors about some types of materials, experimental systems and methods used in many studies. Here, indicate whether each material, system or method listed is relevant to your study. If you are not sure if a list item applies to your research, read the appropriate section before selecting a response.

Materials & experimental systems

n/a	Involved in the study	
☐	☒	Antibodies
☐	☒	Eukaryotic cell lines
☒	☐	Palaeontology and archaeology
☒	☐	Animals and other organisms
☒	☐	Clinical data
☒	☐	Dual use research of concern
☒	☐	Plants

Methods

n/a	Involved in the study	
☒	☐	ChIP-seq
☒	☐	Flow cytometry
☒	☐	MRI-based neuroimaging

Antibodies

Antibodies used

β-actin (Santa Cruz, sc-47778), actin (Sigma-Aldrich, A2103), Flag (Sigma-Aldrich, F1804 or F7425), V5 (Bio-Rad, MCA1360; or Bethyl Laboratories, A190-119A), GST-HRP conjugate (Cyriva, RPN1236), HA (Cell Signaling Technology, 2367 or 3724), IFNAR1 (Abcam, ab124764), JAK1 (BD Biosciences, 610231), TYK2 (Cell Signaling Technology, 14193), phospho-Y1054/1055-TYK2 (Cell Signaling Technology, 68790), PJA2 (Bethyl Laboratories, A302-991A), STAT1 (Santa Cruz, sc-417), phospho-Y701-STAT1 (Cell Signaling Technology, 9167), STAT2 (Santa Cruz, sc-1668), phospho-Y690-STAT2 (Cell Signaling Technology, 88410), MxA (ab143108; gift from Jovan Pavlovic, University of Zurich, Switzerland), IRF9 (BD Biosciences, 610285), DNAJA2 (Abcam, ab157216), KLC2 (Atlas Antibodies, HPA040416), CNOT1 (Cell Signaling Technology, 44613), or IL6ST (Bethyl Laboratories, A304-929A-T), IRDye 800CW Goat anti-Mouse IgG (LI-COR Biosciences, 926-32210), IRDye 800CW Goat anti-Rabbit IgG (LI-COR Biosciences, 926-32211), IRDye 800CW Donkey anti-Goat IgG (LI-COR Biosciences, 925-32214), IRDye 680RD Goat anti-Mouse IgG (LI-COR Biosciences, 926-68070), IRDye 680RD Goat anti-Rabbit IgG (LI-COR Biosciences, 926-68071), HRP-Horse anti-Mouse IgG (Cell Signaling Technology, 7076P2), HRP-Goat anti-Rabbit IgG (Sigma-Aldrich, A0545), or HRP-Rabbit anti-Goat IgG (Sigma-Aldrich, A4174). Antibodies were diluted according to the manufacturers' recommendation.

Validation

Antibodies were used following assessment of the manufacturers' validation statements or, where appropriate and shown in the manuscript, after self-validation by specific gene depletion and assessment of specific loss of corresponding antibody reactivity.

Eukaryotic cell lines

Policy information about [cell lines and Sex and Gender in Research]

Cell line source(s)

Human embryonic kidney (HEK) 293T cells and human lung epithelial A549 cells were both originally from ATCC (CRL-3216 and CCL-185). hTERT-immortalized human lung fibroblasts (MRC-5/hTERT) were a gift from Chris Boutell, University of Glasgow, UK. Primary human bronchial epithelial cells (HBEpCs) were obtained from Epithelix (62-year-old male Hispanic donor; donor 1) and PromoCell (52-year-old female Caucasian donor; donor 2).

Authentication

None of the cell lines were authenticated following acquisition.

Mycoplasma contamination

All cell lines were routinely tested for mycoplasma contamination and tested negative at all times.

Commonly misidentified lines
(See ICLAC register)

None.

Plants

Seed stocks

N/A.

Novel plant genotypes

N/A.

Authentication

N/A.
