# Supplementary material for: Proximal protein landscapes of the type I interferon signaling cascade reveal negative regulation by PJA2
Source: Nat Commun. 2024 May 27;15:4484. doi: 10.1038/s41467-024-48800-5 (PMC11130243; doi:10.1038/s41467-024-48800-5)
Supplement: Supplementary file 4 — Description of Additional Supplementary Files [file 41467_2024_48800_MOESM4_ESM.pdf]

Description of Additional Supplementary Files

File Name: Supplementary Data 1

Description: overview of the different proximity labeling analyses with details on the 103 high-confidence proteins identified in proximity to IFNAR1, IFNAR2, JAK1, TYK2, STAT1, STAT2, and IRF9.

File Name: Supplementary Data 2

Description: overview of the 584 proteins identified in proximity to IFNAR1, IFNAR2, JAK1, TYK2, STAT1, STAT2, and IRF9 with a relaxed threshold of FC ≥ 2.

File Name: Supplementary Data 3

Description: results from the pooled siRNA screen measuring IFN-α2-stimulated antiviral activity against VSV-GFP.

File Name: Supplementary Data 4

Description: analysis of factors identified in proximity to STAT1, STAT2, and IRF9 in both our screen and that of other investigators.
